# On the Hunt: Searching for Poorly Defined Camouflaged Targets

**DOI:** 10.1371/journal.pone.0152502

**Published:** 2016-03-28

**Authors:** Alyssa S. Hess, Andrew J. Wismer, Corey J. Bohil, Mark B. Neider

**Affiliations:** Department of Psychology, University of Central Florida, Orlando, Florida, United States of America; University of Nottingham, UNITED KINGDOM

## Abstract

As camouflaged targets share visual characteristics with the environment within which they are embedded, searchers rarely have access to a perfect visual template of such targets. Instead, they must rely on less specific representations to guide search. Although search for camouflaged and non-specified targets have both received attention in the literature, to date they have not been explored in a combined context. Here we introduce a new paradigm for characterizing behavior during search for camouflaged targets in natural scenes, while also exploring how the fidelity of the target template affects search processes. Search scenes were created from forest images, with targets a distortion (varied size) of that image at a random location. In Experiment 1 a preview of the target was provided; in Experiment 2 there was no preview. No differences were found between experiments on nearly all measures. Generally, reaction times and accuracy improved with familiarity on the task (more so for small targets). Analysis of eye movements indicated that performance benefits were related to improvements in both Search and Target Verification time. Combined, our data suggest that search for camouflaged targets can be improved over a short time-scale, even when targets are poorly defined.

## Introduction

Real world search often involves identifying targets that blend in with their surroundings. Whether it be looking for keys on a messy desk or searching for a camouflaged threat in the field, the process of visual search requires us to properly segment an object from visually similar environmental distractors. Traditional studies of visual search, however, often involve search for targets among distractors of varying similarity on a homogenous background (see [1] for a review). Recent studies have begun to examine the relationships between search behavior and target-background similarity [2, 3]. Consistent with findings that search becomes more difficult as targets and distractors become more similar [4], as a target becomes more similar to the background on which it is superimposed, search performance, as measured by both manual responses and oculomotor behavior, deteriorates in kind. Previously, this has been explained in terms of an inefficient segmentation process for complex backgrounds [5]. Later evidence based on oculomotor measures suggested that individuals search objects that are most salient, rather than focusing on the more subtle, but less salient display regions that were most likely to contain the target. This is somewhat counterintuitive, given that when a target is similar to the background the most salient display regions are the *least* likely to be the target [2]. Interestingly, there is some evidence that performance on these types of search tasks can not only improve with training, but that training does in fact transfer to similar, but novel search displays [6, 7, 8]. While these studies provide a groundwork for the examination of search behavior when targets are camouflaged, most of them used (1) well-defined targets and (2) structured backgrounds, two factors that introduce a gap between what these studies are measuring and what observers are confronted with when engaged in this sort of task in the real world.

When searching for a target in the real world, one could make the case that observers rarely, if ever, possess a perfect representation, or template, of the target they are seeking [9]. Accordingly, it is becoming increasingly argued that search in the real world is guided by non-specific target-templates [10, 11, 12]; I may know I am searching for a stapler, but the internal target representation that I use to guide my search may vary from the target along a number of feature vectors (e.g., color, shape, orientation in space). Along these lines, when participants are asked to search for targets defined at various levels in a categorical hierarchy, targets defined at lower levels of the hierarchy engender the best search performance (e.g., searching for a target defined as “Dessert” vs. searching for a target defined as “Chocolate Ice Cream”) [13]. Presumably, these differences in search are directly related to the quality of the target template; more specific information allows for a better template from which to guide search. Consistent with this notion, recent work [9] suggests that as target templates become less precise, both attentional guidance (as measured by search times) and object identification processes (as measured by verification times) suffer, with higher fidelity templates inducing better performance on these measures. Critically, this distinction between search for well-defined targets and poorly defined targets has not been explored in the context of search for camouflaged targets.

A similarly serious gap in our understanding of search under conditions of camouflage arises from the types of images that have been used in recent studies. Specifically, a number of studies that have characterized search behavior under camouflage conditions have based their inferences on search displays that were not entirely representative of what we find in the real world. Work by Neider and colleagues [2, 6, 8] employed object arrays overlaid on a background constructed systematically (using a tiling method) from a piece of the target. Chen and Hegde [7] used natural textures to form their backgrounds from which observers were asked to search for embedded digital embryos. In both cases, the search tasks were performed on displays that, though perhaps approximating the statistical properties of natural images in some cases, were quite clearly “unscene-like” (also see, [14], for a different but related approach). In the real world, past experiences with a given environment provides us with a wide range of information that might be useful in guiding search, such as learned spatial associations about where targets in a given environment are most likely to appear and item-item co-occurence [15, 16, 17, 18, 19]; see [20] for a review. Generally, while previous studies have provided us with a good understanding of how search processes are affected as targets become more similar to the background on which they appear, and the distractors that they may appear with, it remains unclear whether this understanding reflects similar search tasks conducted in natural scenes (but see, [21] for one example of search for camouflaged targets in a natural environment).

The current studies were designed to build upon our existing understanding of the mechanisms underlying search for camouflaged targets by characterizing search behavior when the target is camouflaged and poorly defined in natural scenes. In two experiments, images of natural forest environments were used to create search scenes; targets were created from a piece of the background. More specifically, targets were made to appear as a slightly distorted piece of the background scene, rather than a recognizable object, and similar to what one might encounter when trying to find a target hidden in a wooded or brush filled area. In Experiment 1, participants were provided with a preview of a target which gave them a perfect visual template from which to guide their search. In Experiment 2, no preview was provided, leaving observers with no specific target template from which to guide search. Size of the search target was also manipulated, with smaller targets resulting in a more difficult search task. Consistent with previous studies by Neider and colleagues [2, 6, 8], we predicted that accuracy and reaction times (RT) would worsen with increasing target difficulty, but that participants might show improvements in those measures as familiarity of the tasks increased (based on training improvements reported in [6, 8, 22]). Additionally, consistent with previous studies of search for well-defined targets compared to categorically defined targets [11, 13], we predicted that overall performance would be better in Experiment 1 when participants were provided with a perfect visual template for each search trial compared to Experiment 2, when participants could not search from a perfect template.

### Experiment 1

In Experiment 1, participants searched natural forest images for the presence of camouflaged targets of varying difficulty in natural scenes. Targets were different on every trial and prior to each trial participants were provided with a preview of the target they would be searching for. To the extent that search for camouflaged targets in natural scenes reflects similar search tasks in non-scenes with structured backgrounds we expected search performance to become worse with increasing difficulty, but to generally improve over the course of the experiment given past findings that search for camouflaged targets is amenable to training [2, 6, 8].

## Method

### Participants

Twenty one participants (7 male, 14 female) from the University of Central Florida (UCF) (age *M* = 19.85, *SD* = 3.31) participated for course credit. All participants had normal or corrected to normal visual acuity (for both near and far sight, measured using a Snellen chart) and color vision (measured with Ishihara plates).

### Apparatus

The experiment was conducted using a Samsung Syncmaster 2233 monitor at a fixed distance of 58cm and subtending 44.1° x 28.3 of visual angle. An EyeLink 1000 eye tracker (SR Research) recorded eye movements during the experiment and button-presses were recorded with a Microsoft Sidewinder gamepad. Gaze measures were derived using the default SR Research algorithms for cognitive research. Eye movements were defined as saccades if they exceeded 1° and either their acceleration reached 8,000°/sec or their velocity reached 30°/sec.

### Stimuli and Design

**Fig 1:**
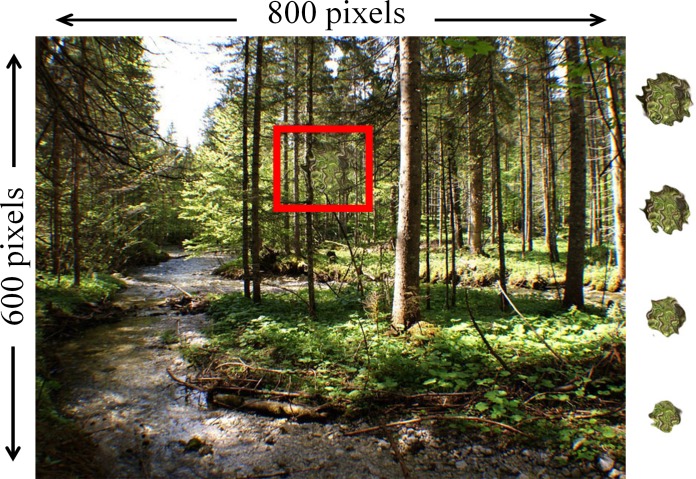
A sample search image containing a 70-pixel target shown inside the red (dark gray) box. The four target sizes in the experiment (70, 60, 50, and 40 pixels, or diameters of 1.9°, 1.7°, 1.4°, and 1.1°, respectively) are illustrated on the right. The targets were different at every location in every image.

**Fig 2:**
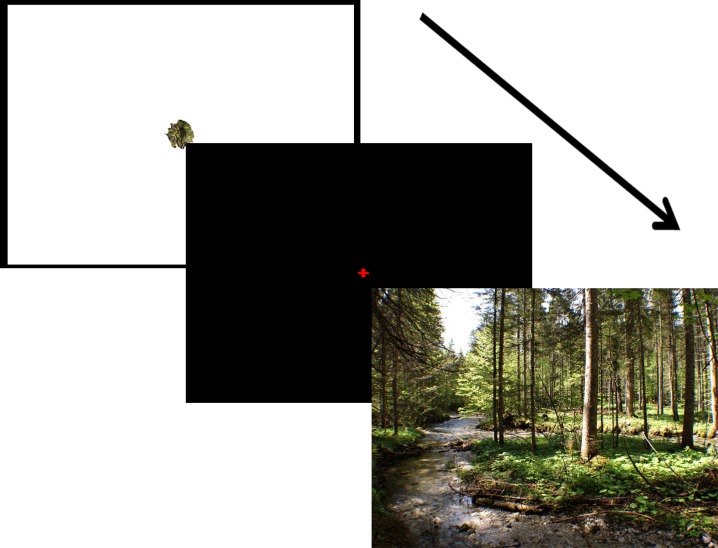
A sample search sequence in Experiment 1. The target was displayed for 1s, followed by a fixation cross for 1s, and then the search scene. In Experiment 2 the target preview was omitted.

The experiment employed a 2 (target present/absent) x 4 (target sizes) repeated measures design. Targets were created by first laying an imaginary grid over each of the 30 800 x 600 pixel natural images to create a 25 cell location array (5 x 5). The center cell was omitted, leaving 24 possible target locations. Four circular selections, varying in size (diameters of 1.9°, 1.7°, 1.4°, and 1.1°, respectively), were then made from the center of each grid location. These selections were then altered by applying a ripple effect at 250% using Adobe PhotoShop (see Fig 1), resulting in a total of 96 possible targets for each image (four per grid location). The ripple effect allowed us to create a set of targets that were highly similar to the search display, but slightly distorted. Essentially, the effect produces a wave-like distortion that slightly repositions image information at a given location without introducing new color information into the image. In target present trials, targets were always placed in the grid location from which they were captured. A sample search scene is displayed in Fig 1, with a graphical depiction of the order of events in the Experiment shown in Fig 2. Participants received eight practice trials to familiarize themselves with the task, followed by four experimental blocks containing 48 search trials (50% target present) each, for a total of 192 search trials. Target size and presence were counterbalanced and interleaved across all blocks. Search images and corresponding targets were selected randomly.

### Ethics Statement

All study protocols and materials were approved by the UCF Institutional Review Board (IRB) (protocol SBE-15-10936) and classified as minimal risk. The UCF IRB requires that studies classified minimal risk obtain informed consent verbally, in order to minimize documentation of identifiable information. Thus, participants provided informed consent verbally to an experimenter prior to participation. The experimenter made note of the verbal informed consent for each participant in de-identified study documents where each participant was assigned a number that was decoupled from their identity.

### Procedure

Participants provided informed consent verbally to an experimenter. They were then screened for normal vision before being read instructions on the experiment. The instructions stipulated that both speed and accuracy were important. Following instructions, participants were calibrated on the Eyelink 1000 using a 9 point calibration procedure. Each participant was re-calibrated prior to each new experimental block. Prior to the experimental trials participants completed 8 practice trials during which they received feedback (no feedback was given during the actual experimental trials).

Trials began with a fixation dot displayed in the center of the screen which participants had to fixate to advance to the actual search task, after which, a 1s preview of the search target was displayed in the center of the screen (in all conditions), followed by a centralized fixation cross for 1s (see Fig 2). The search display then appeared until a button press was entered. Participants were instructed to respond using the gamepad by pressing the left trigger for target present, and the right trigger for target absent. Following the response, the search scene was replaced with the fixation dot, indicating the start of the next trial.

## Results and Discussion

Unless otherwise noted, all analyses reflect repeated measures ANOVA that were conducted on trials in which the participant responded correctly (save for accuracy). Greenhouse-Geisser corrections were applied in cases where the sphericity assumption was violated. Participants were excluded from analyses if they performed at greater than two standard deviations above or below the mean in accuracy or consistently performed at under 50% correct, which represented chance performance. A sub-portion of RT and error data from pilot participants was presented at the Human Factors and Ergonomics Society annual meeting [23].

### Accuracy

**Fig 3:**
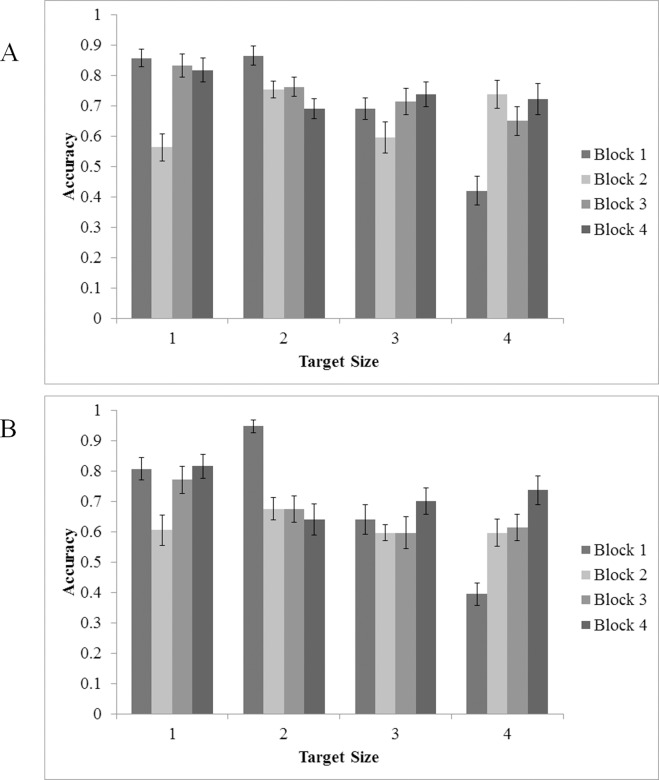
Percent Correct in target present trials as a function of target size (1 = 70 pixel; 2 = 60 pixel; 3 = 50 pixel; 4 = 40 pixel) and experimental block. (A) Experiment 1 and (B) Experiment 2. Error bars indicate one standard error of the mean.

Mean accuracy as a function of target size and block are displayed in Fig 3A. Omnibus ANOVA showed a main effect of target presence, *F*(1,20) = 5.89, *p* < .05, η^2^ = .23, indicating that accuracy varied based on whether or not the target was present. Consistent with typical findings in the search literature, participants were more accurate in target absent trials. There was also a main effect of target size, *F*(2.22, 44.47) = 9.71, *p* < .001, η^2^ = .33; participants were more prone to error as the size of the target decreased. We also found a main effect of trial block, *F*(3, 60) = 4.99, *p* < .01, η^2^ = .20; accuracy increased as participants became more familiar with the task. Interestingly, a significant interaction between target size and block, *F*(9, 180) = 7.64, *p* < .001, η^2^ = .28, appears to reflect that the changes in accuracy over experimental block were largely accounted for by a robust increase in accuracy across blocks at the smallest target size compared to other target sizes, in which accuracy remained relatively stable across blocks. The interaction of target presence and size, *F*(2.06, 41.27) = 3.52, *p* < .05, η^2^ = .15, was also significant, but the interaction of target presence and block was not (*p* = .113). ANOVA also revealed a significant three-way interaction between target presence, size, and block, *F*(9, 180) = 9.93, *p* < .001, η^2^ = .33. Again, analyses were also conducted on the target present and target absent trials separately.

**Fig 4:**
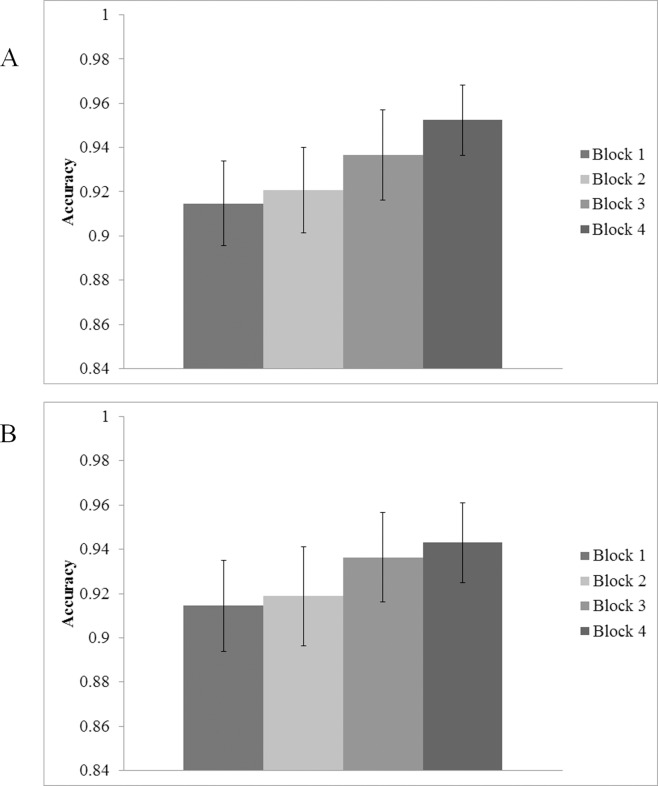
Percent correct in target absent trials as a function of target size (1 = 70 pixel; 2 = 60 pixel; 3 = 50 pixel; 4 = 40 pixel) and experimental block. (A) Experiment 1 and (B) Experiment 2. Error bars indicate one standard error of the mean.

Analysis of target present trials only revealed main effects for target size, *F*(2.14, 42.82) = 7.29, *p* < .005, η^2^ = .27, and block, *F*(2.06, 41.28) = 4.07, *p* < .05, η^2^ = .17, indicating that accuracy varied across difficulty levels and changed over time. Pairwise comparisons for target size indicated poorer accuracy for size 3 compared to sizes 1 and 2, respectively, and size 4 compared to sizes 1 and 2, respectively (ps < .05). Pairwise comparisons for block revealed an initial decrease in accuracy from the first to second block, followed by a significant increase into the third block (all *p*s < .05); block 4 did not differ significantly from block 3. Once again, the two-way interaction between target size and block, *F*(9, 180) = 11.04, *p* < .005, η^2^ = .36, is reflective of robust improvements in accuracy over time at the smallest target size compared to more stable performance over time at the other target sizes. In target absent trials (Fig 4A) there was a main effect of block, *F*(1.99, 39.85) = 13.33, *p* < .001, η^2^ = .40. However, neither the main effect of target size, nor the interaction between target size and experimental block reached significance (all *F* < 3, all *p* > .68).

### Reaction Time

**Fig 5:**
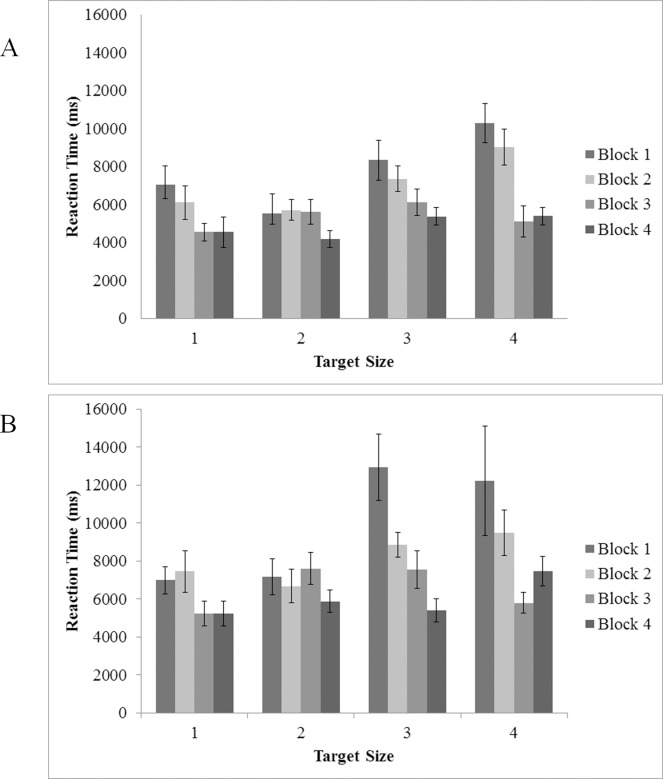
Reaction Time in target present trials as a function of target size (1 = 70 pixel; 2 = 60 pixel; 3 = 50 pixel; 4 = 40 pixel) and experimental block. (A) Experiment 1 and (B) Experiment 2. Error bars indicate one standard error of the mean.

Mean RTs as a function of target size and block are displayed in Fig 5A. Target presence, target size, and experimental block (to assess changes in behavior over time) were submitted to repeated-measures ANOVAs. Overall, we found a main effect of target presence, *F*(1, 20) = 67.42, *p* < .005, η^2^ = .77, indicating that participants took longer to respond in target absent trials. Additionally, main effects of both target size, *F*(3, 60) = 3.77, *p* < .05 η^2^ = .16, and block, *F*(1.48, 29.62) = 24.40, *p* < .001, η^2^ = .55, indicate that RTs generally improved over the course of the experiment and were slower for smaller (more difficult) targets. Significant two way interactions were found between presence and target size, *F*(3, 45) = 4.33, *p* < .01, η^2^ = .22, and presence and block, *F*(1.71, 25.61) = 12.10, *p* < .001, η^2^ = .45. Neither the two-way interaction between target size and block nor the three-way interaction between target presence, size and block (*p*s > .07) reached significance. To better characterize the effects of target size and experimental block, we also analyzed target present and absent trials separately.

**Fig 6:**
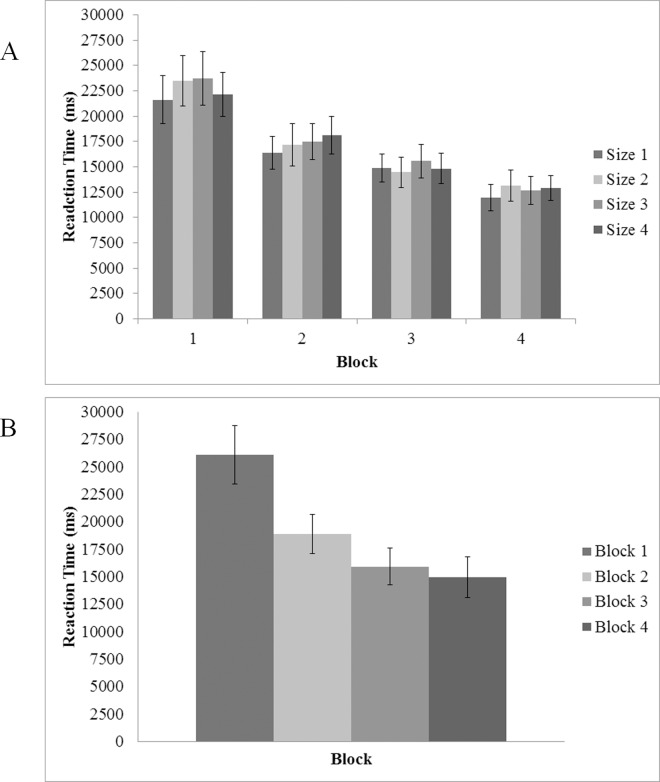
Reaction Time in target absent trials as a function of target size (1 = 70 pixel; 2 = 60 pixel; 3 = 50 pixel; 4 = 40 pixel) and experimental block. (A) Experiment 1 and experimental block only (B) Experiment 2. Error bars indicate one standard error of the mean.

ANOVA analyzing target present trials exclusively indicated a main effect of target size, *F*(3, 45) = 7.82, *p* < .005, η^2^ = .34; pairwise comparisons showed that RTs for sizes 1 and 2 were equivalent (*p* > .05), but both were significantly different from sizes 3 and 4 (*ps* < .05). The two smaller sizes were not significantly different from one another (*p* > .05). A main effect of block in target present trials, *F*(1.93, 28.97) = 16.37, *p* < .005, η^2^ = .52, indicated that performance improved over the course of the experiment. Specifically, pairwise comparisons indicated that participants improved their RTs between blocks 2 and 3, as well as between blocks 3 and 4 (*ps* < .05). Additionally, an interaction between block and target size, *F*(9, 135) = 2.40, *p* < .05, η^2^ = .14, indicated that the pattern of RTs across trial blocks varied as a function of target size; participants showed more robust improvements over time for smaller (i.e., harder) targets compared to larger (i.e., easier) targets. For the smallest target size, RTs improved by 48% from block 1 to block 4 compared to an improvement of 35% for the largest target over the same period. The pattern of data in target absent trials was similar to target present trials. Main effects of both target size, *F*(1.93, 38.54) = 3.68, *p* < .05 η^2^ = .16, and experimental block, *F*(1.46, 29.19) = 25.141, *p* < .001, η^2^ = .557, indicated that participants displayed generally slower RTs as the target became smaller (i.e., more difficult) and that RTs generally became faster as participants became more familiar with task (from block 1 to block 4). The interaction between target size and experimental block was not significant (*p* = .312) (See Fig 6A).

### Scan Path Ratio

Although manual reaction time and error data are informative in conveying differences in aggregate search performance across experimental manipulations, those measures alone do not provide indices of *how* search behavior unfolds over time. Fortunately, eye movement measures can provide such insights. One such measure is scan path ratio, which is calculated by dividing the Euclidean distance of all saccades made in a given trial by the shortest route possible from the center of the screen to the center of the target [24]. The most efficient route possible produces a scan path ratio of 1, with increasing values representing less efficient paths to the target. A benefit of the scan path ratio measure is that it provides an index of oculomotor strategy; a decrease in the ratio (i.e. as the value moves closer to 1) indicates a more efficient scanning strategy. Previously, it has been suggested that improved search strategy is indicative of learning [25, 26]. Because the scan path ratio measure is calculated based on the distance the eye travels before reaching the target, only correct, target present trials were used to calculate the results (no target is present against which to measure in target absent trials.

**Fig 7:**
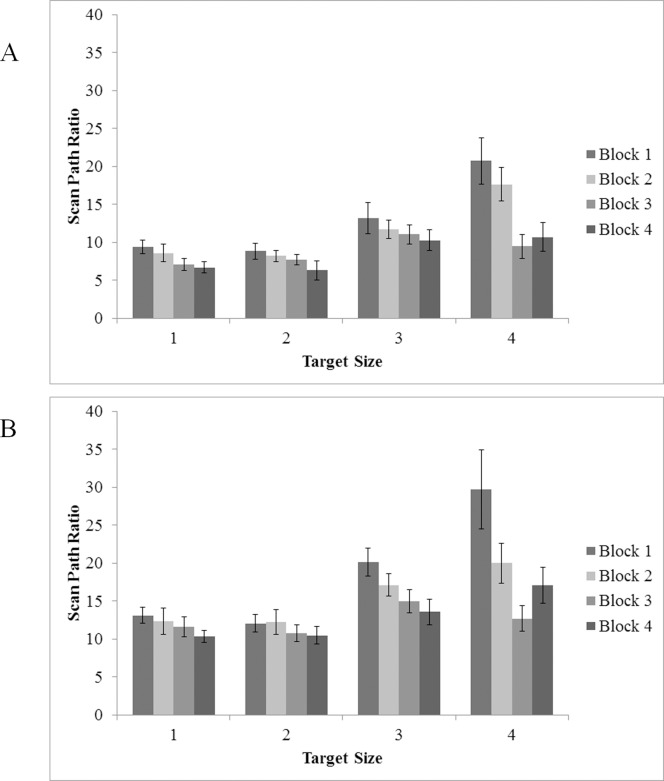
Scan Path Ratio in target present trials as a function of target size (1 = 70 pixel; 2 = 60 pixel; 3 = 50 pixel; 4 = 40 pixel) and experimental block. (A) Experiment 1 and (B) Experiment 2. Error bars indicate one standard error of the mean.

An ANOVA with target size and block as within-subject factors for target present trials was used to characterize changes in scan path ratio across target sizes over time (see Fig 7A). There were main effects of target size, *F*(1.73, 27.72) = 31.28, *p* < .005, η^2^ = .66, and block, *F*(3, 48) = 8.17, *p* < .005, η^2^ = .34; participants adopted more efficient scan paths toward the target as they became more familiar with the task, perhaps reflecting improved tuning of search guidance mechanisms over time. Overall, scan paths were less efficient for smaller (harder) targets (*M* = 16.16, *SE* = 1.72, for smallest target size) than larger (easier) targets (*M* = 8.39, *SE* = .74, for largest target size) (ps < .05). Importantly, a two-way interaction between experimental block and target size (*F*(9, 144) = 3.84, *p* < .005, η^2^ = .20) indicates that improvements in scan path ratio over time were larger for the smaller, more difficult targets than for the larger, less difficult targets (48% improvement for smallest target size compared to 29% improvement for largest target size).

### Search and Verification Time

Our analysis of scan path ratio clearly indicated that participants moved their eyes toward the target more efficiently as they became more familiar with the target. However, it is unclear what the underlying bases for these improvements might have been. On the one hand, guidance mechanisms may have become better tuned to the search targets over time, engendering more efficient paths toward the target. On the other hand, it is also possible that participants became better at sampling (i.e., accepting or rejecting) visual information with practice, and as a result, were able to determine whether an ensemble of visual features at a given location was in fact the target item more quickly. To decouple these two possibilities we further characterized reaction time data in the context of eye movements to provide indices of Search and Target Verification Times. Search Time reflects the time from the onset of the search image to the time in the trial when the participant fixates the target for the first time and provides a measure of search guidance [27]. Verification Time reflects the amount of time that passes from the participant’s initial fixation on the target to when they make a button-press response. Verification Time can be thought of as analogous to a decision making phase during search. Broadly, improvements in guidance mechanisms would be reflected in faster Search Times, and improvements in sampling and decision making processes would be reflected in faster Target Verification Times. It should be noted that these analyses can only be conducted in cases where there is a target in the search image and as such target absent trials are omitted from the analyses.

**Fig 8:**
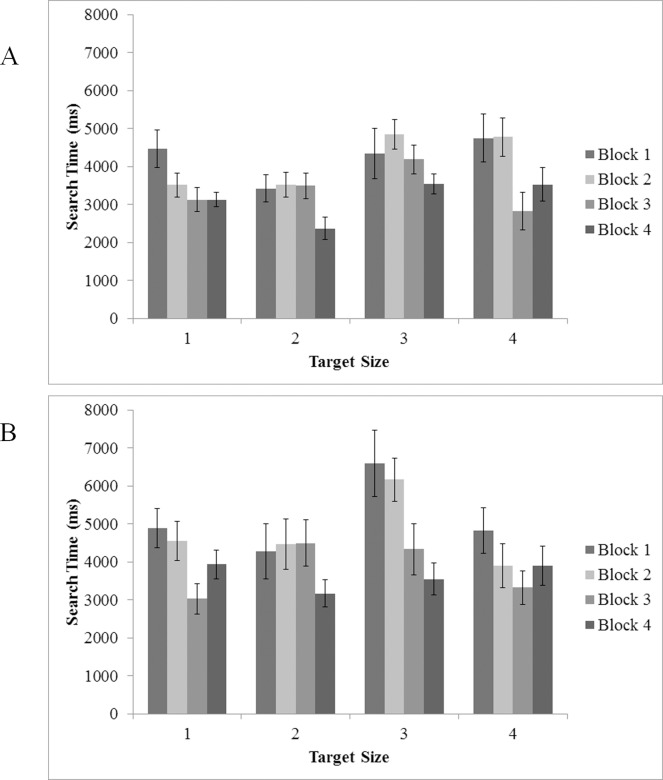
Search Time in target present trials as a function of target size (1 = 70 pixel; 2 = 60 pixel; 3 = 50 pixel; 4 = 40 pixel) and experimental block. (A) Experiment 1 and (B) Experiment 2. Error bars indicate one standard error of the mean.

To assess differences in Search Time over the course of the experiment and across changes in target difficulty, target size and experimental block were submitted to an ANOVA as within-subjects factors for target present trials; results of the analysis are illustrated in Fig 8A. We found main effects of both target size, *F*(3, 48) = 6.95, *p* < .005, η^2^ = .30, and block, *F*(1.84, 29.41) = 8.76, *p* = .001, η^2^ = .35. However, there was no significant interaction between these two factors, *F*(9, 144) = 1.82, *p* = .069, η^2^ = .10. Pairwise comparisons revealed that search times became faster as participants became more familiar with the task, and were generally faster for easier targets (which were statistically equivalent) compared to harder targets (which were statistically equivalent) (*ps* < .05), possibly reflecting more robust search guidance for larger targets and a general improvement in search guidance as participants became more familiar with the task.

**Fig 9:**
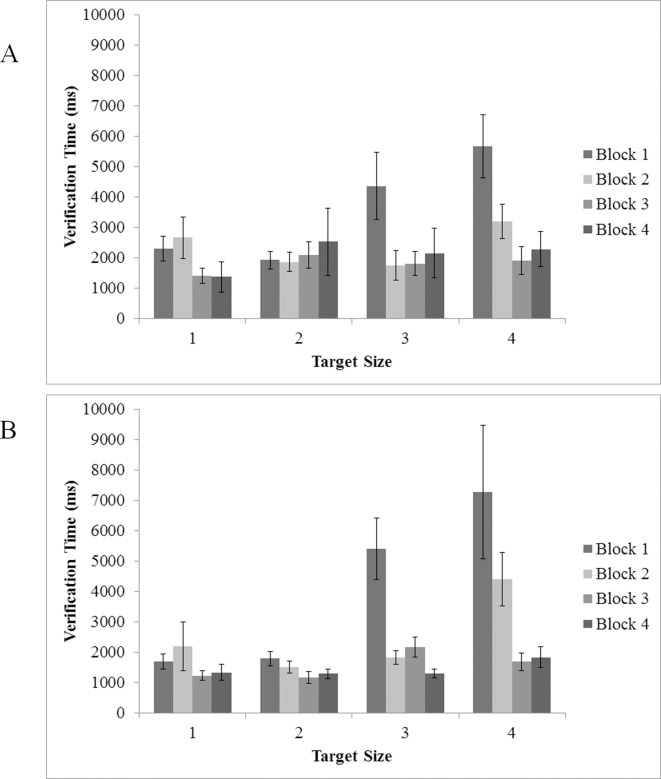
Verification Time in target present trials as a function of target size (1 = 70 pixel; 2 = 60 pixel; 3 = 50 pixel; 4 = 40 pixel) and experimental block. (A) Experiment 1 and (B) Experiment 2. Error bars indicate one standard error of the mean.

An identical analysis was conducted on Verification Time as well (see Fig 9A). A main effect was found for target size, *F*(3, 48) = 6.62, *p* < .005, η^2^ = .29, indicating that Verification Time (time to make a decision) changed with difficulty; larger (easier targets) engendered faster Verification Times. However, there was no main effect of experimental block, *F*(1.66, 26.62) = 2.14, *p* = .144). It is possible that this nonsignificant effect was largely driven by the fairly small improvement in Verification Time at the largest target size (*M* = 2428.46 in the first block, *M* = 1551.46 in the fourth, a difference of 877 ms). In contrast, Verification Times associated with the smallest target size improved robustly from block 1 to block 4 (*M* = 5672.50 in the first block. *M* = 2473.60 in the fourth block, a difference of 3198.90). This interpretation is supported by a significant block x target size interaction, *F*(3.62, 57.95) = 3.82, *p* = .01, η^2^ = .19, indicating that improvements in Verification Time across blocks were larger for smaller (i.e., more difficult targets) than they were for larger (i.e., easier) targets.

### Experiment 2

Our data from Experiment 1 provided validation for our paradigm as a method of exploring visual search for camouflaged targets in natural scenes. Clearly, participants started out quite poorly on the search task, with accuracy levels hovering around chance, thus confirming that the nature of our camouflage manipulation was working as intended. However, and somewhat surprisingly, participants displayed robust improvements in nearly all performance indicators with only modest practice on the task (four experimental blocks), suggesting that training individuals to break camouflage in naturalistic contexts may indeed be an attainable possibility; several studies have indicated success along these lines with more artificial stimuli [6, 8].

Although Experiment 1 provided validation for the experimental paradigm, it did not reflect search for camouflage targets in the real world to the greatest extent possible. More specifically, in Experiment 1 participants were provided with a preview of the target they would be searching for in each trial. In the real world, this is rarely the case; the searcher does not have a perfect representation of the target that needs to be detected. To address this incongruency, in Experiment 2 we had participants search for camouflaged targets in a manner nearly identical to Experiment 1, however, in this case there was no target preview provided. Instead, participants had to form their own strategy for representing the target to inform guidance and sampling processes. Consistent with prior studies [11, 13], we expected that performance would be worse in Experiment 2 than in Experiment 1, however, of primary interest was whether participants would show improvements in search performance over time.

## Method

Nineteen undergraduate students (9 male, 10 female) from the University of Central Florida (age *M* = 20.05, *SD* = 2.57) who were not participants in Experiment 1 participated in Experiment 2 for course credit. The design of the second experiment was identical to the first in all but one aspect. Specifically, in Experiment 2 participants did not receive a target preview before engaging in the search task. Instead, participants were told only to search for a camouflaged target, and were shown its location during practice trials when they could not locate it. As in Experiment 1, participants performed 8 practice trials (4 target present, 4 target absent) while receiving feedback so that they could develop some expectation regarding the nature of the targets. At no time during the Experiment were any targets shown in isolation outside the context of the actual search display.

## Experiment 2 Results and Discussion

All analyses were conducted in a manner similar to Experiment 1. Unless otherwise noted, all analyses were repeated measures ANOVA conducted on trials in which the participant responded correctly. In cases where the assumption of sphericity was violated, a Greenhouse-Geisser correction was applied. Because there was no target preview in Experiment 2, it was only possible to characterize differences in performance associated with target size (i.e., target difficulty) in target present trials (in target absent trials there was neither a target preview nor a search target in the display itself against which effects of target size could be inferred). As a result, data related to target size are only reported for target present trials.

### Accuracy

ANOVA on accuracy showed a significant main effect of presence, *F*(1, 18) = 96.70, *p* < .001, η^2^ = .84, in a pattern consistent with Experiment 1 (see Fig 3B). A main effect of block, *F*(3, 54) = 5.19, *p* < .01, η^2^ = .22, suggested an improvement in accuracy across blocks in both target present and absent conditions. The interaction between target presence and block did not reach significance, *F*(3, 54) = 2.30, *p* = .087, η^2^ = .55.

As in Experiment 1, an ANOVA including target size and block was conducted on target present trials alone, revealing significant main effects of target size, *F*(3, 54) = 24.95, *p* < .005, η^2^ = .58, and block, *F*(3, 54) = 3.93, *p* = .013, η^2^ = .18, and an interaction between target size and block, *F*(9, 162) = 11.40, *p* < .005, η^2^ = .39. Importantly, this interaction reflects the robust improvements in accuracy across experimental blocks in the smallest target condition (improvement from block 1 (40%) to block 4 (74%)) compared to the other target conditions in which accuracy remained fairly stable over the course of the experiment.

An ANOVA analyzing effects in target absent trials indicated there was no significant main effect of block in target absent trials (*p* = .181) (see Fig 4B).

### Reaction Time

Reaction time data are displayed in Fig 5B. An omnibus ANOVA including target presence and experimental block indicated main effects for both target presence, *F*(1, 18) = 78.50, *p* < .005, η^2^ = .8, and block,. *F*(1.37, 24.73) = 14.70, *p* < .001, η^2^ = .45. Participants were quicker to respond in target present trials compared to target absent trials and responses were made more quickly as they became more familiar with the task. The interaction of target presence and experimental block was also significant, *F*(1.42, 25.50) = 14.87, *p* < .001, η^2^ = .45.

ANOVA on target size and experimental block in target present trials only showed main effects of target size, *F*(1.97, 33.45) = 5.36, *p* < .05, η^2^ = .24and block, *F*(1.77, 30.05) = 8.19, *p* < .005, η^2^ = .33. Pairwise comparisons indicated that reaction times for the two largest sizes were equivalent (*ps* > .05), that the smallest size was significantly different from the largest size (*p* < .05) and marginally different from the second largest (*p* = .055). Further pairwise comparisons indicated that significant improvements in reaction time occurred between the first and second half of the experiment (*ps* < .05). Overall, participants were faster to find larger targets than smaller targets and performance improved over the course of the experiment. The interaction between block and target size was not significant, *F*(2.45, 41.72) = 2.71, *p* >.067, η^2^ = .56, indicating that improvements in reaction time over the course of the experiment were independent of target size.

In target absent trials, a main effect of block was also found, *F*(1.307, 23.518) = 16.299,*p* < .001, η^2^ = .48, with pairwise comparisons showing significant improvements in every block (*ps* < .05) save for between blocks 3 and 4 (see Fig 6B).

### Scan Path Ratio

As in Experiment 1, scan path ratios were derived for correct target present trials only; a scan path ratio of 1 represents the most efficient path, in terms of eye movements, to the target, with ratios further from 1 representing increasingly less efficient oculomotor paths to the target. An ANOVA with target size and block as within subject factors was conducted to characterize changes in scan path ratio over time and by difficulty (see Fig 7B). ANOVA indicated a main effect for both target size, *F*(1.72, 27.48) = 17.86, *p* < .005, η^2^ = .53, and block, *F*(1.86, 29.76) = 9.42, *p* = .001, η^2^ = .37. These effects suggest that scan path efficiency changed both over time and by difficulty level; pairwise comparisons indicated paths to the target were less efficient as the target got smaller (size 1 compared to sizes 3 and 4; size 2 compared to sizes 3 and 4), but did improve over the course of the experiment (improvements in each block except from 3 to 4) (*ps* < .05). Critically, there was a significant interaction between block and size, *F*(2.79, 44.59) = 3.50, *p* = .026, η^2^ = .18; scan paths for smaller (i.e., harder) targets improved more (in terms of efficiency) over the course of the experiment than for larger (i.e., easier) targets (21% improvement for the largest target size, 42% improvement for the smallest).

### Search Time and Verification Time

As in Experiment 1, overall reaction times were decomposed into search and verification times in correct target present trials. ANOVA investigating search time with block and target size as within subject variables indicated main effects for both variables target size, *F*(3, 36) = 4.98, *p* = .005, η^2^ = .29, and block, *F*(3, 36) = 5.08, *p* = .005, η^2^ = .30 (see Fig 8B). Overall, participants searched longer for the second to smallest target size, with only slight differences between the other target sizes (all sizes significantly faster than size 3) and search times became faster as the experiment progressed (significantly faster from block 1 to 3, block 1 to 4, and block 2 to 4) (*ps* < .05). Similar to Experiment 1, the interaction between block and target size was not significant (*p* = .310) indicating that differences in search time were consistent across targets and blocks.

A similar ANOVA using target size and block as within subjects variables was conducted on correct target present trials for verification time (see Fig 9B). A main effect was found for target size, *F*(1.27, 15.26) = 5.40, *p* = .028, η^2^ = .31, as well as block, *F*(1.27, 15.27) = 8.12, *p =* .008, η^2^ = .40. Pairwise comparisons indicated that the largest target size was significantly faster than the smallest, and the second largest was significantly faster than both the smaller target sizes (*ps* < .05). Additionally, significant differences were seen between all blocks, save for block 2 to 3 (though there was a ~1000 ms speed improvement with target size collapsed), *ps* < .05. Subjects’ verification time for the two smallest targets decreased rapidly following the first block, following the pattern of Experiment 1. A marginally significant interaction between block and target size, *F*(1.83, 22.01) = 3.43, *p* = .054, η^2^ = .22, reflects the differential improvements over blocks in verification time across target size. More specifically, verification times for the two smaller targets decreased profoundly over the course of the experiment (~76% and 75% improvement from block 1 to block 4 for target sizes 3 and 4, respectively), whereas verification times for larger targets remained relatively stable.

## Comparing Experiments 1 and 2

The data from Experiment 2 were remarkably similar, pattern-wise, to those of Experiment 1. Generally, after initially poor performance at the start of the experiment, participants showed robust improvements in nearly every performance indicator over only four experimental blocks. Interestingly, that participants were able to perform this difficult search task so well in the absence of a target preview suggests that perceptual representations, at least for the sort of abstract targets used here, can be formed quite quickly during visual search and used efficiently in the service of even very difficult search tasks. Our findings raised an interesting question: is there a cost or benefit associated with a perfect target template in this sort of difficult camouflage search task where targets are rather “unobject-like”. To answer this question we compared the data from Experiment 2 directly to those of Experiment 1 treating Experiment as between-subject factor. Due to the nature of the target absent trials in Experiment 2 (i.e., they could not be linked to a particular target), comparisons between Experiments 1 and 2 were only conducted on target present trials.

### Accuracy

Again, experiment, block, and target size were submitted to ANOVA (see Fig 3). A main effect was found for target size, *F*(2.04, 36.74) = 27.62, *p* < .005, η^2^ = .61, as well as block, *F*(3, 54) = 5.22, *p* = .003, η^2^ = .23. There was also a two-way interaction between target size and block, *F*(9, 162) = 23.13, *p* < .005, η^2^ = .56. This indicates that accuracy changed over time based on target size when experiments are collapsed. The marginally significant three-way interaction between experiment, block, and target size, *F*(9, 162) = 1.91, *p* = .054, η^2^ = .10, likely reflects the fact that participants in Experiment 2 started much more poorly than those in Experiment 1, specifically in the smaller target sizes, but by block 3, had generally leveled out, and by the final block, were performing at accuracy equivalent to that of the Experiment 1 participants (*M* block 4 in Experiment 1 = 74%, *M* block 4 in Experiment 2 = 73%). All other main effects and interactions with Experiment as a factor did not approach significance, all *F* < 1, all *p* > .44.

### Reaction Time

An omnibus ANOVA was conducted on target present trials only, using experiment as a between-subjects factor, and block, and target size as within-subjects factors (see Fig 4). The ANOVA indicated a main effect of size, *F*(3, 42) = 8.09, *p* < .005, η^2^ = .37, and block, *F*(2.09, 29.22) = 24.44, *p* < .005, η^2^ = .64. Additionally, a two-way interaction was found between block and target size, *F*(3.55, 49.71) = 4.54, *p*< .005, η^2^ = .25. Critically, no main effects or interactions were found for the Experiment factor, meaning that performance in the context of reaction time was equivalent across Experiments 1 and 2 (all *F* < 4, all *p* > .09); participants were able to respond to the presence of a target at the same speed regardless of whether or not they were provided with a perfect visual target template.

### Scan Path Ratio

Because the scan path ratio measure is dependent on the presence of a target in the search display, data were again only analyzed in correct target present trials (see Fig 7). An ANOVA including experiment, target size, and block indicated main effects for experiment, *F*(1, 12) = 10.17, *p* = .008, η^2^ = .46, size, *F*(1.73, 20.78) = 40.19, *p* < .005, η^2^ = .77, and block, *F*(3, 36) = 10.33, *p* < .005, η^2^ = .46. Of most interest is the main effect of Experiment which reflects less efficient scan path ratios in Experiment 2 compared to Experiment 1. Although visual examination of the data suggest that this effect may have been driven by the two smallest (most difficult) target sizes, the Experiment x target size interaction was not significant, nor were any other interactions (all *F* < 1, all *p* > .43) save for that of block and size, *F*(3.13, 37.50) = 5.31, *p* = .003, η^2^ = .31. It should be noted that despite the fact that scan path ratios were less efficient in Experiment 2 relative to Experiment 1, as previously indicated, this difference did not translate into differences in overall reaction time, which was statistically equivalent across experiments.

### Search Time and Verification Time

An ANOVA analyzing search time conducted on correct target present trials using experiment, target size, and block as factors indicated main effects for target size, *F*(3, 27) = 7.64, *p* < .005, η^2^ = .46, and block, *F*(3, 27) = 10.57, *p* < .005, η^2^ = .54 (see Fig 8). Critically, all effects related to experiment were non-significant (all *F* < 2, all *p* > .21); regardless of whether or not they were provided with a visual target template, took similar amounts of time to initially fixate the target. Interestingly, this lack of a difference suggests that guidance mechanisms may not necessarily benefit from a specific prototype in this sort of camouflage search task.

To examine differences in verification time associated with the availability of a visual target template, an ANOVA was conducted with experiment, block, and target size as factors in correct target present trials (See Fig 9). Similar to the analysis of Search Time, main effects were found for target size, *F*(1.76, 15.80) = 6.19, *p* < .05, η^2^ = .41, and block, *F*(1.41, 12.70) = 8.12, *p* < .05, η^2^ = .47, but no effects associated with the experiment factor reached significance, all *F* < 2, all *p* > .34. Again, the presence or absence of visual target template had little effect on the time required to respond to the target once it was fixated. No other effects reached significance (all *p* < .05).

## General Discussion

Our main goals in the current studies were to (1) characterize visual search behavior in the context of camouflaged targets in natural scenes, (2) to develop a novel paradigm from which future work might investigate possible training benefits in search for camouflaged targets, and (3) to do so in a manner representing what is typically encountered in the real world, where the searcher is almost always unaware of the specific visual properties of the target item. We achieved goals 1 and 2. Our data from Experiment 1 indicated that participants required more time to locate a camouflaged target in our natural forest images as the target became smaller (i.e. more difficult); a finding broadly consistent with previous studies of search for camouflaged targets that did not use natural scenes [2, 6, 8]. Interestingly, examination of the reaction time data over the course of the experiment revealed robust improvements in performance from block 1 to block 4, particularly when participants searched for the hardest targets (nearly 50% improvement in reaction time for target size 4 over the course of the experiment). The reaction time improvements were coupled with either flat or improving accuracy rates, indicating that time-based benefits were not the byproduct of a speed accuracy tradeoff. Analysis of eye movement data suggested that these reaction time improvements were subserved by improvements in both search guidance and decision making mechanisms (improvements in both Search and Verification Times). The latter finding is consistent with a number of previous studies [2, 22] and may suggest that with increased task familiarity comes an improvement in segmentation processes. With practice, observers are better able to extract target-related information from the background region. Underlying mechanisms notwithstanding, the finding that participant performance improved dramatically after only four blocks of trials provides grounds for optimism that our camouflage search paradigm may be a viable means for training camouflaged target detection in real world contexts.

The extent to which we achieved goal 3 is less clear. In Experiment 2 we had participants search for the same targets in the same images as Experiment 1, however, they did not receive a visual preview of the target. Instead, participants were simply asked to search for irregularities and then left to develop their own knowledge representation of the target items. Performance in Experiment 2 was nearly identical to that in Experiment 1; reaction time, accuracy, and most gaze measures (with scan path ratio being the one exception) were similar regardless of whether or not participants had prior access to a visual template of the target. This finding is of critical importance given that in the real world we rarely, if ever, have a perfect representation of the target we are searching for. A recent study by Hout and Goldinger [9] demonstrated that increasing the imprecision of a target template produces impairments in visual search at both the guidance and target identification levels. This is particularly relevant in the context of search for camouflaged targets. The purpose of camouflage is to conceal an object, animal, or person so that they blend in with their surroundings. As such, if one were to search for a “person” prototype (which in of itself is different from all studies in the visual search domain in which a target preview is provided) the result would likely be quite poor. Instead, when searching for a camouflaged target, an observer is probably better served looking for some inconsistency in the environment, and here-in lies the reason for which our data here must be interpreted with a degree of caution. To some degree, it is unclear to what extent observers in Experiment 2 needed to rely on an acquired representation of the target item at all. It is possible that observers simply searched for some “anomaly” in the display. In its strictest form, such a strategy would not require any reliance on a target template. Overall, Experiment 2 suggests that, broadly speaking, observers are able to search for abstract targets (or patterns), even when they are undefined visually, with little to no cost in overall performance measures and that they can learn to do so rapidly during the course of visual search. It does not, however, tell us definitively whether search under such conditions is predicated on some cumulative representation of the target class acquired over time or a simple strategy of searching for something “different.” Further characterizing the factors underlying the pattern of performance we observed in Experiment 2 remains an interesting question for future studies to consider.

Returning to more practical applications associated with improving camouflage detection skills in various operational settings, an important question that will need to be answered beyond that of prolonged training improvement is the extent to which performance improvements in our task transfer to similar, but novel tasks and contexts. Though we cannot provide a definitive answer to the question given the current data, based on data we do have, we have good reason to believe that transfer of training is likely (see, [6, 8] for demonstrations of transfer of training in camouflage search in simple search arrays) and this belief is based largely on the eye movement data we have presented here. Specifically, it can be inferred that to the extent that the improvements in performance that participants displayed in our task are associated with improved scan strategies, those improvements are likely to transfer to novel circumstances, such as searching for novel classes of camouflaged targets in novel environments. However, if the improvements are solely predicated on participants learning target-specific information over time, then performance improvements are less likely to transfer. Although these competing possibilities are not entirely mutually exclusive, our eye movement measures provide indices of both via Search and Verification Times. Improvements in the former over blocks indicates that participants learned to scan the display more efficiently and would provide strong evidence that learning in the task is likely to transfer to novel tasks, whereas improvements in the latter would suggest that learning is target specific. As our data from Experiments 1 and 2 clearly indicated, participants showed robust improvements in Search Time over experimental blocks at all target sizes, supporting the idea that participants were learning to scan the search display faster with practice. Participants also showed improvements in Verification time as well. Taken together, our data suggest that while participants may be learning some target-specific information over time that may be aiding broader search processes, it seems unlikely that this learning alone is what’s underlying time-based improvements on the task. We will explore questions related to transfer of training in our camouflage search task in future studies.
